# Prenatal Exposure to Metals Is Associated with Placental Decelerated Epigenetic Gestational Age in a Sex-Dependent Manner in Infants Born Extremely Preterm

**DOI:** 10.3390/cells14040306

**Published:** 2025-02-18

**Authors:** Katelyn K. Huff, Kyle R. Roell, Lauren A. Eaves, Thomas Michael O’Shea, Rebecca C. Fry

**Affiliations:** 1 Curriculum in Toxicology and Environmental Medicine, University of North Carolina at Chapel Hill, Chapel Hill, NC 27514, USA; huff25@live.unc.edu; 2 Institute for Environmental Health Solutions, University of North Carolina at Chapel Hill, Chapel Hill, NC 27514, USA; kyle.roell@unc.edu (K.R.R.); laeaves@live.unc.edu (L.A.E.); 3 Department of Environmental Sciences and Engineering, University of North Carolina at Chapel Hill, Chapel Hill, NC 27514, USA; 4 Department of Pediatrics, University of North Carolina at Chapel Hill, Chapel Hill, NC 27514, USA; moshea52@email.unc.edu

**Keywords:** epigenetic gestational age, DNA methylation, placenta, manganese, copper

## Abstract

Prenatal exposure to metals can influence fetal programming via DNA methylation and has been linked to adverse birth outcomes and long-term consequences. Epigenetic clocks estimate the biological age of a given tissue based on DNA methylation and are potential health biomarkers. This study leveraged the Extremely Low Gestational Age Newborn (ELGAN) study (*n* = 265) to evaluate associations between umbilical cord tissue concentrations of 11 metals as single exposures as well as mixtures in relation to (1) placental epigenetic gestational age acceleration (eGAA) and the (2) methylation status of the Robust Placental Clock (RPC) CpGs. Linear mixed effect regression models were stratified by infant sex. Both copper (Cu) and manganese (Mn) were significantly associated with a decelerated placental eGA of −0.98 (95% confidence interval (CI): −1.89, −0.07) and −0.90 weeks (95% CI: −1.78, −0.01), respectively, in male infants. Cu and Mn levels were also associated with methylation at RPC CpGs within genes related to processes including energy homeostasis and inflammatory response in placenta. Overall, these findings suggest that prenatal exposures to Cu and Mn impact placental eGAA in a sex-dependent manner in ELGANs, and future work could examine eGAA as a potential mechanism mediating in utero metal exposures and later life consequences.

## 1. Introduction

Environmental contamination from metals and metalloids is a widespread public health concern [1]. For instance, the U.S. Agency of Toxic Substance and Disease Registry (ATSDR) classified arsenic, lead, and mercury among the highest ranking hazardous substances on the 2022 Substance Priority List [2]. Inorganic arsenic remains a major contaminant of groundwater on a global scale and is a known carcinogen [3,4]. This is especially alarming for those whose source of drinking water is unregulated private wells [5]. On the other hand, trace amounts of essential metals such as zinc and manganese are needed to maintain normal biological processes and functions. However, deficiencies or excess accumulations of these essential trace elements from chronic environmental and occupational exposures may have detrimental health effects including neurological [6,7] and metabolic disorders [8,9]. Pregnant people and the developing fetus are particularly vulnerable to such chemical exposures, with impacts on pregnancy complications [10,11] and birth outcomes [12].

The placenta is a key organ during pregnancy that acts as the protective interface between the fetus and the pregnant person by regulating the transfer of specific molecules such as nutrients and antibodies. Maintaining its health and function is critical for proper fetal development. Several metals including mercury and lead can cross the placental barrier and reach the fetus, while others such as cadmium accumulate in placental tissue [13,14,15]. In utero exposures and stressors during critical windows of development can lead to epigenetic adaptions that impact health outcomes later in life as postulated under the Developmental Origins of Health and Disease (DOHaD) hypothesis [16,17]. While prior work has established relationships between prenatal exposures to metals/metalloids and placental DNA methylation [18,19], the role of epigenetic gestational aging (eGA) has yet to be fully explored as a potential mechanism. Epigenetic gestational clocks utilize machine learning algorithms to estimate biological aging based upon DNA methylation levels in perinatal tissues such as placenta or umbilical cord blood [20,21,22,23]. While some chemical exposures have been investigated with eGA derived from cord blood [24,25], there is a gap regarding eGA in the placenta, which has been shown to offer distinct biological aging implications compared to other perinatal tissues [26].

Four major epigenetic clock algorithms have been established using placental tissue. The first was proposed by Mayne et al. which estimated eGA using methylation levels at 62 CpGs and accurately predicted the gestational age in their validation dataset (*r* = 0.95, *p* < 2.2 × 10^−16^) [23]. While this placental clock was trained on CpGs in the Infinium Human Methylation27 (27K) and Methylation450 (450K) BeadChip arrays, Lee et al. utilized methylation data from both 450K and Infinium Human MethylationEPIC (EPIC) platforms in 1102 placental samples to develop three more placental clock algorithms: (1) the Robust Placental Clock (RPC), (2) the Control Placental Clock (CPC), and (3) the refined RPC (rRPC) [22]. Each of these clocks are more suitable for specific types of data. The RPC, comprising 558 CpGs, is largely unaffected by pregnancy complications; the CPC, comprising 543 CpGs, is intended for uncomplicated pregnancies; and the rRPC, comprising 395 CpGs, is intended for uncomplicated term pregnancies. Notably, Lee et al. showed that the RPC outperformed Mayne et al.’s placental epigenetic clock in predicting gestational age, especially for placental samples from pregnancies complicated by conditions such as preeclampsia and gestational diabetes [22].

Elucidating the impacts of early exposures to trace elements on eGA at birth may provide an additional understanding of their potential short- and long-term consequences for the fetus. Specifically, in this study, we hypothesized that essential metals may be linked to placental eGA deceleration, while non-essential metals could lead to eGA acceleration. We leveraged the Extremely Low for Gestational Age Newborn (ELGAN) study to evaluate associations between prenatal exposure to 11 individual metals—indicated by their umbilical cord concentrations—and placental eGAA estimated using the RPC. Furthermore, since these metals often co-occur naturally in the environment and may interact antagonistically or synergistically, quantile g-computation was also used to evaluate associations between metal mixtures and eGAA.

## 2. Materials and Methods

### 2.1. The ELGAN Study

This study includes secondary analyses utilizing data from the Extremely Low for Gestational Age Newborn (ELGAN) study, which is a prospective cohort study originally aimed to understand potential drivers of developmental and brain abnormalities in preterm infants as detailed elsewhere [27]. In short, this study recruited mothers who gave birth before 28 weeks of gestation at one of 14 participating study sites across 5 U.S. states from 2002 to 2004. Each site followed Institutional Review Board (IRB)-approved protocols, and the participating mothers provided informed consent after hospital admission either before or shortly after delivery. This resulted in an original cohort of 1506 infants and 1249 mothers. It is worth noting that while pregnant people can encompass non-binary, intersex, and transgender communities, we refer to birthing parents as “mothers” in this study to remain consistent with the language used during data collection.

**Figure 1:**
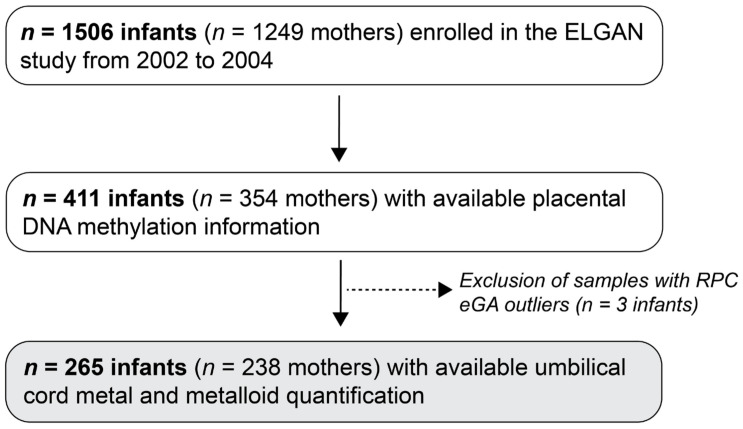
Study sample selection. This flowchart outlines inclusion and exclusion criteria applied to the ELGAN cohort to yield the final analytic sample for this study.

The ELGAN cohort is a unique resource based on its rich -OMICs data including placental DNA methylation, allowing for the estimation of placental epigenetic gestational age, as well as its assessment of fetal exposure to metals using umbilical cord tissue. Placental and umbilical cord tissue samples were collected following birth and stored for future analyses. Specifically, CpG analysis and the measurement of metals in umbilical cord samples were assessed after 2015. Placental DNA methylation quantification was performed on samples from participants who remained enrolled in the study by age 10 and who had available placental tissue of sufficient quality, limiting the sample to 411 infants from 354 mothers. Among these participants, 268 infants (241 mothers) also had umbilical cord tissue that underwent assessment for select essential and non-essential trace elements. Following the exclusion of potential outliers as further discussed below, the final analytic sample consisted of 265 infants from 238 mothers (Figure 1).

### 2.2. Chronologcial Gestational Age Ascertainment

Ascertainment of chronological gestational age in the ELGAN study was based on a tiered hierarchical approach of available data as previously reported [28]. Briefly, dates of embryo retrieval, intrauterine insemination, or ultrasound prior to 14 weeks of gestation were prioritized for gestational age determination. When these data were unavailable, ascertainments were based on the following methods with descending preference: (1) ultrasounds performed at or after the 14th week of gestation, (2) menstrual dating without ultrasound confirmation, and (3) gestational age estimates recorded at the neonatal intensive care unit.

### 2.3. Placental Sampling and DNA Methylation Quantification

Placental sampling and quantification of DNA methylation have previously been described in depth in prior studies [29,30]. In summary, placentas were placed in a sterilized basin immediately after delivery. An approximately one-gram tissue sample was isolated from the base of the exposed chorion of each placenta, placed in sterile cryovials, and frozen immediately in liquid nitrogen. Specimen were then stored at −80 °C for future analysis. For DNA extraction, placental segments of ~0.2 g were sectioned from each sample and rinsed in sterile 1× phosphate-buffered saline (PBS) (Fisher Scientific, Waltham, MA, USA) to remove residual blood. Segments were held on dry ice before being homogenized in Buffer RLT with β-mercaptoethanol (Qiagen, Valencia, CA, USA). Following the manufacturer’s protocols, AllPrep DNA/RNA/miRNA Universal Kits (Qiagen, Valencia, CA, USA) were used to extract DNA sequences of greater than 18 nucleotides from the homogenized samples. For DNA methylation quantification, bisulfite conversion was performed on extracted DNA using EZ DNA Methylation Kits (Zymo Research, Irvine, CA, USA), and the Infinium MethylationEPIC BeadChip (Illumina, San Diego, CA, USA) assessed methylation status across more than 850,000 CpGs. The ratio of methylated to unmethylated signal intensities at a given CpG represented the average methylation status or β value. Data underwent background correction via the normal-exponential out-of-band (noob) correction method [31] and functional normalization [32]. Further, principal component analysis (PCA) was used to evaluate potential batch effects. Since plate position was identified as a source of variation, data were corrected utilizing the ComBat function in the surrogate variable analysis (sva) package in R [33].

### 2.4. Placental Epigenetic Gestational Age Acceleration Estimations

Placental epigenetic gestational age (eGA) was estimated for each ELGAN using the RPC that was developed by Lee et al. [22]. This clock algorithm comprises 558 CpGs that were previously selected using the elastic net regression of gestational age on DNA CpG methylation. To generate epigenetic gestational age acceleration (eGAA), eGA estimates were regressed onto chronological GAs. Positive residuals from these models were considered acceleration while negative values were interpreted as deceleration. Note that three of the placental samples in this study yielded residuals that were greater than three standard deviations from the mean; thus, these were considered potential outliers and excluded from the final analytic sample.

Since the RPC is trained on placental samples derived from normal as well as complicated pregnancies ranging from 5 to 42 gestational weeks, the RPC is ‘robust’ to adverse pregnancy conditions such as preeclampsia that may occur more prevalently in the extremely preterm cohort used in this study. Our previous study by Clark et al. demonstrated that the chronological GA was correlated with RPC-estimated eGA (*r* = 0.61) in the ELGAN placental subset of 408 [30]. In the present study, there was a moderate correlation of 0.59 (*p* < 0.01) with a median absolute error (MAE) of 2.04 in the full analytic sample (Supplementary Figure S2). When stratified by fetal sex, male GA was more strongly correlated with eGA (*r* = 0.69, *p* < 0.01, MAE = 1.99) compared to that of females (*r* = 0.41, *p* < 0.01, MAE = 2.12).

### 2.5. Quantification of Metals and Metalloids in Umbilical Cord Tissue

Umbilical cord tissue collection and processing for metals/metalloids concentrations utilized standard protocols outlined in detail in prior studies [34,35]. Shortly following delivery, two 1 cm segments of umbilical cord tissue were collected using a sterile technique. These samples were then immediately placed in cryovials that were submerged in liquid nitrogen and stored at −80 °C until they were later transported to the Wadesworth Center to undergo analysis for trace elements.

Here, eight metals (barium (Ba), cadmium (Cd), copper (Cu), lead (Pb), manganese (Mn), mercury (Hg), strontium (Sr), and zinc (Zn)) and three metalloids (arsenic (As), antimony (Sb), and selenium (Se)) were quantified. While metalloids possess both metal and nonmetal properties, for increased readability and concise communication, the term ‘metals’ is used to collectively refer to all 11 metals and metalloids in this study. Once umbilical cord tissue samples were delivered to the Wadsworth Center, they were accessioned according to standard laboratory procedures and were intermediately stored at −80 °C. Samples were thawed and cut into segments of ~50 mg by dry mass using high-purity tantalum tools created in-house to avoid potential contamination with other trace elements in stainless steel. To remove any superficial blood, all samples were washed with double-deionized (DDI) water (Thermo Scientific Barnstead, Waltham, MA, USA) and then deposited in a 13 mL acid-washed tube. Samples were freeze-dried to constant mass using a slow five-step program for complete water removal. Next, they were digested in concentrated double-distilled HNO_3_ using a Microwave-Assisted Reaction System (MARS) with Xpress vessels by the “One-Touch Animal Tissue” method and then diluted to ~10 g in DDI water and held at 4 °C. Using a method that was optimized for the analysis of umbilical cord tissue, digested samples were analyzed with an Agilent 8900 Inductively Coupled Plasma tandem Mass Spectrometer (ICP-MS/MS) equipped with an SPS4 autosampler and Octopole Reaction System (ORS), with axial acceleration technology. The method was further validated with four National Institute of Standards and Technology (NIST) Standard Reference Materials (SRMs) as follows: NBS 1577 Bovine Liver, NIST 1577b and 1577c Bovine Liver, and NIST 8414 Bovine Muscle Powder. These SRMs underwent the same method of freeze-drying, digestion, and analysis as the umbilical cord samples. Values for sample spikes, duplicates, blanks, and calibrators were carefully monitored during the study. Note that recovery values for spikes and duplicates were within ±20%. The method limits of detection (LOD) for each selected metal in this study are as follows: Cu (0.074 μg/g), Mn (0.010 μg/g), Se (0.10 μg/g), Zn (1.4 μg/g), Sb (0.88 ng/g), As (0.42 ng/g), Ba (8.5 ng/g), Cd (0.32 ng/g), Hg (0.79 ng/g), Pb (2.6 ng/g), and Sr (0.039 μg/g). All 265 samples had detectable Cu, Mn, Se, Zn, As, Ba, Pb, and Sr levels, while 95%, 98%, and 97% of samples had detectable Sb, Cd, and Hg concentrations, respectively. For the samples that were at or below detection, concentrations were imputed as LOD divided by √2 [36].

### 2.6. Covariate Selection

To select potential confounders for adjustment, a direct acyclic graph (DAG) approach was used (Supplementary Figure S1). The minimally sufficient adjustment set included the following covariates: (1) pre-pregnancy body mass index (BMI) (continuous); (2) maternal age (continuous, years); (3) smoking exposure while pregnant (no/yes); (4) parity (0, 1, 2+); (5) public health insurance (no/yes); and (6) maternal educational attainment (high school diploma or less, some college, college degree or greater). Placental cell composition was also included as a potential confounder since prior studies have suggested placental cell type may influence in utero transfer of metals and eGAA [26,37]. The planet package was employed to estimate the cell composition of placental samples [38]. Note that since births spanned 23 to 28 weeks of gestation, the placental reference libraries of the first and third trimesters were averaged to estimate the cell type variation in the second trimester [30]. Following principal component analysis (PCA), the first two eigenvectors, PC1 and PC2, were found to represent a majority of the cell-type variation and were thus included as covariates in ongoing analyses.

### 2.7. Statistical Analysis

Descriptive statistics for the 11 cord metals were calculated for the study’s full analytic sample. Since each metal exhibited a right-skewed distribution, all metal concentrations underwent log-transformation to normalize data used in subsequent statistical analyses.

For individual trace elements, linear mixed modeling was used to evaluate the associations between concentrations of each metal (exposure) and placental eGAA (outcome). Mixed effects were included to account for multiple siblings born to the same mother. These models were also adjusted for the key covariates previously listed (i.e., pre-pregnancy BMI, maternal age, smoking, parity, insurance, education, placental cell type). To address potential sex-specific associations, models was stratified by infant sex assigned at birth (female/male). Statistical significance was defined as *p* < 0.05. Furthermore, to elucidate potential molecular mechanisms underlying significant eGAA, we additionally examined associations between concentrations of select cord metals and the CpG methylation status of the individual RPC 558 CpG loci. For each CpG, linear mixed regression was run, adjusting for the same covariate set and stratifying by fetal sex. These resulting *p*-values were adjusted using the Benjamini and Hochberg procedure to correct for multiple comparisons and statistical significance was set as *q* < 0.1.

Prior work has shown that most cord metal concentrations in the ELGAN study are positively correlated with each other [34,35]. This is true for our analytic sample as well, with 42 out of the 55 total metal pairs exhibiting significant Spearman’s rank correlations (Supplementary Table S1). Given the high correlations between exposures to these metals, we sought to examine associations between exposure to metal mixtures and placental eGAA through quantile-based g-computation using the qgcomp package in R [39,40]. This method estimates the overall mixture effect on placental eGAA of simultaneously increasing all exposures by one quartile. Note that the analytic sample used for mixtures’ analyses was reduced to *n* = 238 after randomly selecting one sample from multiples born to the same mother. Linear regression models were run utilizing the qgcomp.noboot function and adjusting for the same confounders used in the individual metal analyses. Quantile-based g-computation also estimates weights for each of the metals included in the model which indicates the relative proportion each metal contributes to the overall effect. These weights can be positive or negative, and their absolute values sum up to one in each respective direction. In addition to a total mixtures model, we also evaluated mixtures of (1) essential metals (Cu, Mn, Se, and Zn) and (2) non-essential metals (As, Ba, Cd, Hg, Pb, Sb, and Sr) in respect to placental eGAA. In addition to adjusting for the same set of seven confounders, the essential metal mixture model included seven non-essential metals while the non-essential metal mixture model included four essential metals to account for potential confounding from co-exposures.

Finally, geographical location can influence the exposure to metals. Specifically in the ELGAN study, the majority of the metals differed by study site regions as shown in Supplementary Table S2. Thus, additional sensitivity analyses were run in which models for single metals and metal mixtures were stratified by three regional groups categorizing the 14 study sites in the ELGAN study: New England, North Carolina, and the Midwest. The New England region encompasses Baystate Children’s Hospital, Boston Children’s Hospital (i.e., Beth Israel Deaconess Medical Center, Brigham and Women’s Hospital and Massachusetts General Hospital), Tufts Medical Center, UMass Memorial Hospital, and Yale-New Haven Children’s Hospital. The North Carolina region included East Carolina University, the University of North Carolina at Chapel Hill, and Wake Forest Baptist Medical Center. The Midwest region included Helen DeVos Children’s Hospital, Michigan State University and Sparrow Hospital, the University of Chicago Medical Center, and Beaumont Children’s Hospital. All statistical analyses were performed in R (version 4.3.1) [41].

## 3. Results

### 3.1. Study Population

After applying the exclusion/inclusion criteria, this study yielded a final sample size of 265 mother–infant dyads, with almost half (45.28%) of the sample being from the New England region (Table 1). The majority of participants self-identified as non-Hispanic (91.70%) and White (62.98%), followed by Black (28.24%) and Other (8.78%). The median age was 29.40 years, and the median pre-pregnancy BMI was 23.19. Most participants had at least some college education or greater (61.15%); did not receive public insurance (67.18%); and did not smoke during pregnancy (91.57%). This sample consisted of slightly more male (54.82%) than female infants (45.28%). The median gestational age was 26.29 weeks, while the median epigenetic gestational age (eGA) estimated by the RPC was 28.17 weeks. Following the regression of the eGA on chronological gestational age, the median eGA acceleration residual was −0.03 weeks.

While the distribution of metals examined in ELGANs has been reported in prior studies [34,35], concentrations of the 11 metals examined in this study’s analytic sample are shown in Table 2. Overall, Zn and Cu exhibited the highest median concentrations in ELGAN umbilical cord tissue at an average of 59.90 μg/g and 3.48 μg/g, respectively. In contrast, the least abundant metals quantified in the cord tissue included Sr and Cd with median levels of 0.55 ng/g and 1.28 ng/g, respectively. While concentrations also differed by fetal sex for select metals, none reached statistical significance (*p* > 0.05). For example, males exhibited higher median Pb (*p* = 0.06; female: 14.45 ng/g; males: 17.80 ng/g) and Cu (*p* = 0.08; females: 3.59 μg/g; males: 3.42 μg/g) levels compared to females.

### 3.2. Individual Umbilical Cord Metal Levels and Placental eGAA

**Figure 2:**
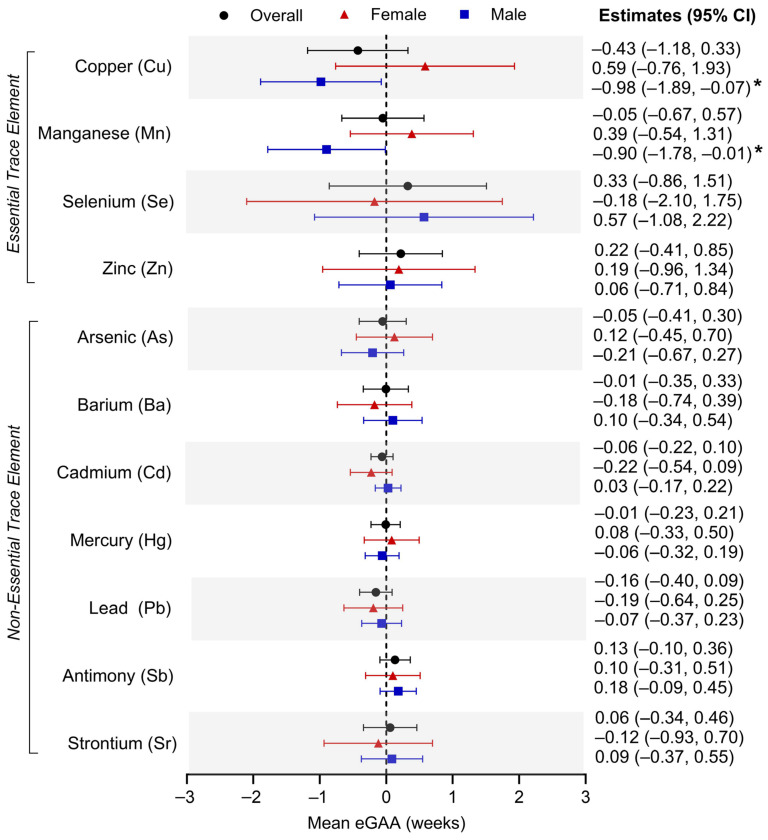
Associations between individual prenatal metal exposure and placental epigenetic gestational age acceleration (eGAA) in the overall ELGAN sample and infant sex-stratified groups. * *p* < 0.05.

Linear mixed modeling was used to evaluate the relationship between prenatal exposure to each of the 11 metals of interest and placental eGAA (Figure 2). Adjusting for the aforementioned covariates, we did not observe significant associations between individual exposures to any of the metals and eGAA in the overall sample. When stratified by infant sex, no significant associations were found in female infants. However, umbilical cord Cu concentration was associated with an average eGA deceleration of −0.98 weeks of gestation (95% CI: −1.89, −0.07) in male infants. Additionally, prenatal exposure to Mn exhibited a male-specific association with eGA deceleration (adjusted β: −0.90 weeks of gestation; 95% CI: −1.78, −0.01).

Supplementary Figure S3 further evaluates these relationships when exposures were sorted into quartiles and highlights sex-specific effects for Cu and Mn. Compared to those in the first quartile, those in the fourth quartile for Cu umbilical cord concentrations in male infants displayed an average eGA deceleration of −0.42 weeks (95% CI: −0.77, −0.08). Additionally, while not reaching statistical significance, Cu concentrations in the second (adjusted β: −0.28 weeks of gestation; 95% CI: −0.64, 0.09) and third quartiles (adjusted β: -0.17 weeks of gestation; −0.52, 0.18) trended with eGA deceleration. This inverse U-shaped relationship between Cu concentrations and eGAA in males contrasts with the U-shaped trend observed in female infants (quartile 2 = adjusted β: 0.58 weeks of gestation; 95% CI: 0.14, 1.02; quartile 3 = adjusted β: −0.08 weeks of gestation; 95% CI: −0.53, 0.37; quartile 4 = adjusted β: 0.44 weeks of gestation; 95% CI: −0.04, 0.92). Furthermore, increasing quartiles of Mn trended with decelerated eGA in male infants (quartile 2 = adjusted β: 0.02 weeks of gestation; 95% CI: −0.35, 0.38; quartile 3 = adjusted β: −0.08 weeks of gestation; 95% CI: −0.44, 0.27; quartile 4 = adjusted β: −0.28 weeks of gestation; 95% CI: −0.63, 0.07), suggesting a negative monotonic dose-response. Contrastingly, females exhibited positive linear trends between Mn and eGAA (quartile 2 = adjusted β: −0.46 weeks of gestation; 95% CI: −0.95, 0.02; quartile 3 = adjusted β: −0.24 weeks of gestation; 95% CI: 0.75, 0.26; quartile 4 = adjusted β: −0.01 weeks of gestation; 95% CI: −0.50, 0.48).

### 3.3. Individual Umbilical Cord Metal Levels and RPC CpG Methylation

To investigate potential biological pathways related to specific RPC CpGs that underlie the observed associations, we also ran linear mixed regression models to evaluate associations between the 11 umbilical cord metals and methylation levels at the 558 CpGs that comprise the RPC. Based upon the individual metal analyses and focusing on male-specific effects for prenatal Mn and Cu, we found that Cu concentrations were significantly (*p* < 0.05) associated with methylation at 21 RPC CpG sites (annotating to 18 genes), while Mn concentrations were associated with methylation at 25 RPC CpGs (annotating to 20 genes) (Table 3). After multiple testing corrections, prenatal Mn retained significant associations (*q* < 0.10) with methylation levels at the following four RPC CpGs: cg25298189/AT-rich interactive domain 3A (*ARID3A*) (β: 1.22; 95% CI: 0.41, 2.04); cg16673477/CREB-regulated transcription coactivator 1 (*CRTC1*) (adjusted β: −0.95 weeks of gestation; 95% CI: −1.59. −0.32); cg14620572/adhesion G protein-coupled receptor D2 (*GPR144*) (adjusted β: −1.98 weeks of gestation; 95% CI: −3.32. −0.65); and cg03836586/neurexophilin 1 (*NXPH1*) (adjusted β: −1.62 weeks of gestation; 95% CI: −2.71, −0.52). Notably, *ARID3A* plays a role in regulating inflammatory response in the placenta, while both *CRTC1* and *NXPH1* have been implicated in neurological function in the brain [42,43,44]. Additionally, prenatal Cu conserved the significant association with methylation at the RPC CpG probe cg14654324, annotating to retinoid x receptor alpha (*RXRA*) (adjusted β: 1.56 weeks of gestation; 95% CI: 0.58, 2.53). Specifically, this gene is involved in the regulation of fatty acid homeostasis and cellular differentiation during placental development [45,46].

### 3.4. Umbilical Cord Metal Mixtures and Placental eGAA

Utilizing quantile g-computation, increasing concentrations of all 11 prenatal metals was not significantly associated with placental eGA acceleration or deceleration in the overall population (adjusted β: −0.02 weeks of gestation; 95% CI: −0.23, 0.19) or when stratified by female (adjusted β: −0.07 weeks of gestation; 95% CI: −0.45, 0.31) or male (adjusted β: −0.03 weeks of gestation; 95% CI: −0.28, 0.22) infants (Table 4). We also did not observe associations in the overall or sex-stratified populations when metals were separated into essential (Cu, Mn, Se, and Zn) and non-essential (Sb, As, Ba, Cd, Pb, Hg, and Sr) trace element groups. However, while they did not reach statistical significance, there were eGA deceleration trends in male-specific analyses for the essential metals sub-group (adjusted β: −0.07 weeks of gestation; 95% CI: −0.29, 0.16) with Mn and Cu having the greatest negative contributions of −0.52 and −0.48, respectively. The non-essential metals sub-group exhibited a positive trend with eGAA in males (adjusted β: 0.12 weeks of gestation; 95% CI: −0.13, 0.37), while eGA deceleration was observed in females (adjusted β: −0.04 weeks of gestation; 95% CI: −0.34, 0.26). Although they yielded inverse effects, Ba and Pb weighted the non-essential metals mixture model the greatest in both male (Ba: 0.46; Pb: 0.36) and female (Ba: −0.56; −0.33) infants.

### 3.5. Sensitivity Analyses by Study Site Region

To account for geographical variation in metal exposure, individual metal and metal mixture analyses were stratified by the New England, North Carolina, and Midwest regions. We did not observe significant associations between any of the single metals and placental eGAA across any of the three regions (Supplementary Table S3). When evaluating metal mixtures using quantile g-computation by region, the mixture model comprising all 11 metals was significantly associated with an eGA deceleration of −0.30 weeks of gestation (95% CI: −0.59, −0.01) for the New England region (Supplementary Table S4). Among the metals assigned to the negative weightings, Mn (−0.26), Cu (−0.25), and As (−0.22) had the largest contributions.

## 4. Discussion

The present study evaluated the relationships between metal levels in umbilical cord tissue and placental eGAA in the ELGAN study. Overall, there were two main findings. First, the relationships between the individual essential metal concentrations and eGAA at birth were sex dependent. Specifically, levels of Cu and Mn were associated with decelerated eGA in male infants only. Second, both prenatal Cu and Mn levels in males were associated with methylation at RPC CpGs. Specifically, Cu was associated with DNA methylation in *RXRA* which regulates placental energy homeostasis and cellular differentiation, while Mn was associated with DNA methylation in *ARID3A*, *CRTC1*, *GPR144*, and *NXPH1*, which have been implicated in inflammatory and neurocognitive processes.

Prenatal Cu and Mn were associated with eGA deceleration in male but not in female infants. This finding was particularly interesting since the metals did not display sex-based differences in their accumulation in ELGAN cord tissue and were comparable to Cu and Mn cord levels reported in other studies [47,48]. Previous research has also shown sex-specific effects for prenatal and early-life exposures to these metals in relation to development and late-life outcomes [49,50]. The observed sex-specific relationships in this study were further accentuated when exposures were analyzed within quartiles, revealing opposite dose-responses between eGAA estimates and increasing Cu and Mn quartiles in males and females. A potential biological mechanism underlying these sex-specific effects may include sexually dimorphic DNA methylation patterns that have previously been observed in placenta, with males showing higher levels of methylation in autosomal regions [29,51]. Sex differences have also been shown among associations between gestational age and placental CpG methylation in which males exhibited 15 times as many CpGs associated with gestational age as females [52]. These distinctions could contribute to differences in related placental signaling and homeostasis. Overall, our data suggest a unique vulnerability of male infants to the metal–eGA association. These results may also have implications for later-in-life health where males who are born extremely preterm are at increased risk for adverse outcomes [53,54]; thus, additional research is required to elucidate potential mechanisms underlying sex differences for eGAA at birth and the later-in-life consequences.

Prior studies have shown associations between exposure to single metals and metal mixtures and epigenetic aging in adults [55,56,57]. Specifically, Xiao et al. and Boyer et al. both reported inverse relationships between exposure to mixtures of essential metals such as Se and Zn and Horvath, GrimAge, and DunedinPACE epigenetic age acceleration metrics [56,57]. However, due to the high tissue specificity of the epigenetic clock algorithms [26], it is difficult to draw direct comparisons across different studies looking at aging using adult and perinatal tissues. To date, one other study has examined the relationship between prenatal metal exposure and epigenetic aging at birth and early in life. Bozack et al. utilized DNA methylation measured in cord blood and mid-childhood blood samples from a prospective U.S. cohort to estimate both Bohlin eGA and Horvath epigenetic age metrics [25]. They reported negative linear associations between single essential metals—specifically Cu, Mn, and Zn—and Horvath epigenetic age acceleration at birth (Cu: −0.96 weeks (95% CI: −1.90, −0.02), Mn: −0.94 weeks (95% CI: −1.83, −0.05), and Zn: −1.19 weeks (95% CI: −2.09, −0.29)) [25]. Exploratory factor analysis further demonstrated the metal mixture with the highest loadings from essential metals (i.e., magnesium (Mg), Cu, Se, and Zn) associated with a 1.01 week (95% CI: −1.89, −0.13) deceleration in Horvath epigenetic age at birth [25]. While performed in cord blood samples, both results support our findings that prenatal exposure to essential metals may influence epigenetic gestational aging early in the infant’s life.

Cu and Mn can be obtained through diet and are necessary for proper bodily function in humans, especially for maintaining health during pregnancy and fetal growth. Cu from the birthing parent can cross the placenta via high-affinity Cu transporter CTR1 and ATPases ATP7A/B [58]. It acts as an antioxidant and is required for connective tissue development metabolic homeostasis; however, too low or high Cu concentrations can increase the risk of detrimental outcomes including hypochromic anemia and gestational diabetes, respectively [59]. In this study, Cu levels measured in umbilical cords were associated with CpG methylation at 21 out of the 558 RPC CpGs in male infants, which annotated to 18 genes including *RXRA*. RXRA is a nuclear receptor that participates in placental development through regulating fatty acid transport and the secretion of essential hormones that support trophoblast differentiation during gestation [45,46]. Prior work has also shown altered PPARα/RXRα activation in male placentas from spontaneous preterm birth [60]. While there have been other reports of placental Cu levels and differential DNA methylation [61], *RXRA* and retinoic acid signaling has not previously been implicated with Cu exposure. Further research is required to understand how this signaling may facilitate differential epigenetic aging in the placenta in relation to Cu exposure.

While Mn is important for cellular metabolism, bone development, and the mitigation of oxidative stress, excess levels can lead to adverse effects including neurotoxicity [7,62,63]. Due to increased demand during pregnancy, blood concentrations of Mn in the birthing parent increase during pregnancy, with the highest levels observed in cord blood [64]. Gestational Mn is actively transported across the placenta and can cross the blood–brain barrier of developing fetuses [65,66]. Abnormal Mn levels during pregnancy can negatively impact birth and fetal outcomes such as preeclampsia [67,68] and infant birthweight [69,70] as well as later childhood consequences such as higher odds of elevated blood pressure in children aged 6 to 12 years old [71].

In the present study, exposure to Mn levels in the umbilical cords of male infants was also associated with CpG methylation at 25 RPC CpGs that annotate to 20 genes. Specifically, Mn concentrations were associated with a higher methylation status at cg25298189 which annotates within the gene body of *ARID3A*. ARID3A is a transcription factor and was previously reported to be essential for placentation by promoting the development of the trophectoderm and regulating inflammatory response [72,73]. Given recent work by Grundeken et al., showing that prenatal exposure to Mn is positively associated with placental weight and mitochondrial DNA copy number [74], future studies could examine whether the epigenetic dysregulation of *ARID3A* may serve as a potential mechanism for the impact of Mn on placental health and aging.

We observed significant associations between exposure to Mn and lower placental methylation levels at cg16673477 and cg03836586 located within the gene bodies of *CRTC1* and *NXPH1*, respectively. While less is known about the functions of these genes in the placenta, *NXPH1* and *CRTC1* play significant roles in the brain and could impact early neurodevelopmental programming via the placenta–brain axis [75]. Specifically, NXPH1 regulates synaptic plasticity [42], while CRTC1 has been linked to aging-related disorders such as Alzheimer’s disease [76] and regulates gene expression related to dendritic maturation and inflammation to promote blood–brain barrier integrity, memory, and learning [43,44,77]. Interestingly, exposure to high concentrations of Mn is also associated with increased neurotoxicity via β-amyloid-plaque upregulation, reactive oxygen species generation, and the disruption of neurotransmitter homeostasis leading to neurodegenerative disease states [78]. More research is required to understand whether the placental epigenetic clock may mediate the relationship between prenatal Mn and later infant health.

This study is unique in its evaluation of the relationship between in utero exposure to metals and placental eGAA and adds to the limited literature connecting reproductive toxicology and eGAA. In addition, this research highlights potential mechanisms underlying observed relationships by examining the relationships between exposure and methylation levels at the individual CpGs of the RPC. These data reinforce the importance of the placenta as a target tissue that can be used to assess what we term as the “Placental Origins of Health and Disease.” However, this study is not without limitations. First, the ELGAN study is comprised only of infants born extremely premature (i.e., less than 28 weeks of gestation), all born after one or more pregnancy complications; thus, the results of the present study may not be directly generalizable to term pregnancies. To address this limitation and potential confounding bias, we opted to utilize the RPC to estimate eGAA since this placental epigenetic clock algorithm was specifically trained to be unaffected by complicated pregnancies [22]. Second, this study lacked information surrounding maternal dietary information. This is important as certain foods such as seafood may influence metal exposure levels [79,80]. Third, while eGA was estimated in placenta, exposures to metals were quantified in umbilical cord tissue which is distinct and typically understudied compared to other perinatal biospecimens. Prior studies found strong correlations between metal concentrations in cord tissue, cord blood, and placenta for metals including Hg and Se but no correlations for others including Zn [47,81]. Thus, some metals may exhibit different concentrations depending on the type of perinatal tissue and should be considered when comparing studies and interpreting results. Fourth, metal quantification in cord tissue was only feasible at one point (i.e., at delivery) in this study so timing-specific effects cannot be inferred. Further, present-day exposure levels of some metals such as Pb may differ from exposure levels to which mothers of ELGAN participants were exposed; however, the investigation of epigenetic processes such as placental eGA can provide insights into how early-life stressors can impact later-in-life health outcomes as posited by the DOHaD hypothesis. Finally, when quantifying metals in cord tissue, speciation analysis was not possible in this study. For instance, while total As was quantified, inorganic As (iAs) is generally considered more toxic than organic forms, with arsenite (iAsIII) being more toxic than arsenate (iAsV) [82]. Overall, the interpretation of effects may be influenced by varying proportions of different chemical species for a given metal.

## 5. Conclusions

The present study is among the first to examine the association between metal concentrations in umbilical cords and placental eGAA using both individual and mixture-based approaches. We found that exposure to Mn and Cu was associated with lower mean eGA in male infants. Furthermore, these metals also exhibited associations with methylation status at several RPC CpG sites which were within genes related to placental fatty acid transport and inflammation as well as neurological development. These results bring awareness to metal exposures during pregnancy and their potential implications on child health outcomes, further emphasizing the importance of monitoring levels of environmental contaminants. In relation to clinical implications of this work, it is unlikely that clinicians will use the umbilical cord as an indicator of prenatal exposure to toxicants. However, these data highlight the need for environmental and/or biomonitoring which could be achieved through the testing of water samples as well as the collection and testing of urine/serum prior to and throughout pregnancy [83]. Future research should evaluate trimester-specific dose responses between prenatal metal concentrations and placental eGAA as well as potential epigenetic mechanisms underlying the sex-specific results. Additional work should investigate whether accelerated or decelerated eGA mediates the relationships between in utero exposures to Mn and neurodegenerative disease risk later in life.

## Figures and Tables

**Table 1:** Demographics of the study’s analytic sample in the Extremely Low Gestational Age Newborn (ELGAN) study (*n* = 265).

Maternal Characteristics	Median (IQR) or *n* (%)
Age, years	29.40 (24.70, 34.00)
Pre-pregnancy body mass index (BMI)	23.19 (20.49, 29.27)
Self-reported race	
White	165 (62.98%)
Black	74 (28.24%)
Other	23 (8.78%)
Missing	3
Self-reported ethnicity	
Non-Hispanic	243 (91.70%)
Hispanic	22 (8.30%)
Educational attainment	
High school diploma or less	101 (38.85%)
At least some college	55 (21.15%)
College degree or greater	104 (40.00%)
Missing	5
Parity	
0	147 (55.47%)
1	74 (27.92%)
2+	44 (16.60%)
Smoking during pregnancy	
No	239 (91.57%)
Yes	22 (8.43%)
Missing	4
Public insurance	
No	176 (67.18%)
Yes	86 (32.82%)
Missing	3
Study site region	
New England	120 (45.28%)
North Carolina	81 (30.57%)
Midwest	64 (24.15%)
**Infant Characteristics**	**Median (IQR) or *n* (%)**
Gestational age, weeks	26.29 (25.14, 27.29)
Epigenetic gestational age (eGA), weeks	28.17 (27.05, 29.32)
eGA acceleration (eGAA) residual, weeks	−0.03 (−0.52, 0.48)
Sex	
Female	120 (45.28%)
Male	145 (54.82%)
Birthweight, grams	
≤750	101 (38.11%)
751–1000	116 (43.77%)
1001–1250	40 (15.09%)
>1250	8 (3.02%)

IQR, Interquartile range from 25th percentile to 75th percentile.

**Table 2:** Distributions of umbilical cord metal concentrations in the full ELGAN study analytic sample (*n* = 265), the female-only subset (*n* = 120), and the male-only subset (*n* = 145).

Metal	Unit	Sample	Mean (SD)	Range	Median (IQR)	*p* ^1^
Copper (Cu)	μg/g	Full	3.83 (1.37)	1.62–14.31	3.48 (2.97, 4.32)	0.08
		Female	3.67 (1.15)	1.96–7.93	3.42 (2.88, 4.09)	
		Male	3.96 (1.52)	1.62–14.31	3.59 (3.02, 4.53)	
Manganese (Mn)	μg/g	Full	0.40 (0.37)	0.10–5.58	0.34 (0.30, 0.42)	>0.90
		Female	0.43 (0.51)	0.10–5.58	0.34 (0.30, 0.42)	
		Male	0.38 (0.18)	0.19–1.70	0.34 (0.30, 0.42)	
Selenium (Se)	μg/g	Full	0.88 (0.18)	0.44–1.98	0.86 (0.78, 0.97)	0.80
		Female	0.87 (0.18)	0.44–1.61	0.87 (0.77, 0.97)	
		Male	0.89 (0.17)	0.44–1.98	0.85 (0.79, 0.96)	
Zinc (Zn)	μg/g	Full	69.22 (44.40)	29.87–487.50	59.90 (52.20, 71.50)	0.13
		Female	66.70 (41.72)	29.87–445.50	57.40 (51.00, 69.90)	
		Male	71.31 (46.54)	33.40–487.50	61.50 (53.80, 71.70)	
Antimony (Sb)	ng/g	Full	8.30 (27.53)	0.62–360.93	3.09 (1.80, 5.71)	>0.90
		Female	8.47 (34.05)	0.62–360.93	3.13 (1.76, 5.60)	
		Male	8.17 (20.77)	0.62–149.21	3.08 (1.85, 5.93)	
Arsenic (As)	ng/g	Full	7.20 (9.44)	1.26–77.70	4.70 (3.31, 7.48)	0.30
		Female	7.44 (11.67)	1.26–77.70	4.58 (3.32, 6.76)	
		Male	7.00 (7.13)	1.28–66.84	4.73 (3.30, 8.11)	
Barium (Ba)	ng/g	Full	117.97 (207.75)	17.50–3131.90	81.90 (56.40, 128.40)	>0.90
		Female	127.77 (288.90)	19.80–3131.90	87.05 (58.58, 124.45)	
		Male	109.85 (100.05)	17.50–789.30	81.00 (56.40, 131.00)	
Cadmium (Cd)	ng/g	Full	24.05 (253.35)	0.23–4057.21	1.28 (0.75, 2.86)	0.20
		Female	5.01 (12.06)	0.23–74.77	1.20 (0.71, 2.29)	
		Male	39.80 (342.06)	0.23–4057.21	1.33 (0.80, 3.24)	
Lead (Pb)	ng/g	Full	32.95 (67.79)	2.60–708.70	16.10 (9.30, 29.90)	0.06
		Female	26.73 (47.11)	2.60–350.20	14.45 (8.83, 25.83)	
		Male	38.10 (80.82)	2.90–708.70	17.80 (10.60, 33.60)	
Mercury (Hg)	ng/g	Full	14.67 (19.09)	0.56–174.11	8.13 (3.63, 17.53)	0.20
		Female	12.07 (13.97)	0.56–93.36	7.46 (3.35, 15.07)	
		Male	16.82 (22.29)	0.56–174.11	8.51 (4.02, 20.20)	
Strontium (Sr)	μg/g	Full	0.70 (0.48)	0.17–3.33	0.55 (0.39, 0.86)	0.60
		Female	0.65 (0.43)	0.19–3.33	0.55 (0.41, 0.79)	
		Male	0.74 (0.53)	0.17–3.00	0.56 (0.38, 0.90)	

SD, standard deviation; IQR, interquartile range from the 25th percentile to 75th percentile; ^1^ Wilcoxon rank sum test comparing median metal concentrations between females and males.

**Table 3:** Top associations (*p* < 0.05) between prenatal copper and manganese exposures and RPC CpG methylation in male ELGAN infants.

Prenatal Copper (Cu): 21 RPC CpGs						
CpG Probe	Chromosome	Genomic Region ^a^	Gene	β (95% CI) ^b^	*p*	*q* ^c^
cg14654324	9	Body	*RXRA*	1.56 (0.58, 2.53)	0.00	0.03 *
cg15909725	11			−1.35 (−2.4, −0.3)	0.01	0.12
cg01958723	20			−0.64 (−1.16, −0.13)	0.02	0.14
cg08493294	2	Body	*DNMT3A*	0.82 (0.15, 1.5)	0.02	0.15
cg15488978	10	Body	*C10orf41*	−1.36 (−2.48, −0.24)	0.02	0.15
cg22947322	17	Body	*IGF2BP1*	0.54 (0.09, 0.99)	0.02	0.16
cg00035630	11	TSS1500	*DEPDC7*	−0.95 (−1.75, −0.16)	0.02	0.17
cg20327163	15	Body;5′UTR	*NR2F2*	−0.6 (−1.1, −0.09)	0.02	0.17
cg10482057	6	Body	*KIAA0319*	−0.93 (−1.72, −0.15)	0.02	0.17
cg15862128	14	TSS200	*MIR494*	−0.91 (−1.75, −0.07)	0.03	0.25
cg18752527	2	Body	*HECW2*	0.85 (0.07, 1.64)	0.03	0.25
cg12551957	7	Body	*FBXL18*	0.78 (0.06, 1.51)	0.03	0.26
cg14415214	11	1stExon	*PHLDA2*	−0.97 (−1.88, −0.06)	0.04	0.26
cg21467614	6	1stExon	*TNF*	−0.62 (−1.2, −0.04)	0.04	0.27
cg24244478	11	Body	*MAP6*	−0.59 (−1.14, −0.03)	0.04	0.27
cg07634706	16	TSS200	*CCL17*	−0.68 (−1.34, −0.03)	0.04	0.28
cg22755932	15	Body	*ARRDC4*	−0.94 (−1.84, −0.04)	0.04	0.29
cg02388882	10	5′UTR	*AGAP11*	−1.64 (−3.25, −0.03)	0.05	0.31
cg02603128	9	Body	*FRMD3*	0.79 (0.01, 1.57)	0.05	0.32
cg12077698	1	Body	*TRIM62*	−0.56 (−1.11, 0)	0.05	0.32
cg03543893	20			−1.52 (−3.02, −0.01)	0.05	0.32
**Prenatal Manganese (Mn): 25 RPC CpGs**						
**CpG Probe**	**Chromosome**	**Genomic Region**	**Gene**	**β (95% CI)**	***p***	***q***
cg25298189	19	Body	*ARID3A*	1.22 (0.41, 2.04)	0.00	0.04 *
cg16673477	19	Body	*CRTC1*	−0.95 (−1.59, −0.32)	0.00	0.04 *
cg14620572	9	Body	*GPR144*	−1.98 (−3.32, −0.65)	0.00	0.04 *
cg03836586	7	Body	*NXPH1*	−1.62 (−2.71, −0.52)	0.00	0.05 *
cg26502666	2	5′UTR	*SPDYA*	−2.08 (−3.65, −0.5)	0.01	0.10
cg17452301	10	Body	*PWWP2B*	0.95 (0.21, 1.7)	0.01	0.12
cg08493294	2	Body	*DNMT3A*	0.8 (0.16, 1.43)	0.02	0.14
cg02012338	4	Body	*CYP4V2*	0.55 (0.1, 0.99)	0.02	0.14
cg21523908	1	TSS200	*FAM5B*	−1.45 (−2.63, −0.27)	0.02	0.14
cg22193385	12	Body	*KRT7*	1.47 (0.27, 2.66)	0.02	0.15
cg09163779	10	1stExon;5′UTR	*RPP30*	−0.57 (−1.05, −0.1)	0.02	0.16
cg01152073	5	5′UTR;TSS1500	*SQSTM1*	0.71 (0.12, 1.3)	0.02	0.16
cg12816936	3			0.46 (0.07, 0.85)	0.02	0.17
cg04474049	7	TSS1500	*CAV1*	0.64 (0.1, 1.17)	0.02	0.17
cg23044186	5			0.64 (0.1, 1.19)	0.02	0.18
cg16970628	12	Body	*TMEM132C*	−0.69 (−1.3, −0.08)	0.03	0.21
cg26140475	8			0.51 (0.05, 0.97)	0.03	0.23
cg04110105	3	5′UTR;1stExon	*CCR2*	1.07 (0.08, 2.05)	0.03	0.25
cg14789214	10	Body	*KCNIP2*	0.5 (0.03, 0.96)	0.04	0.27
cg15405432	5	TSS200	*FLJ44606*	−1.7 (−3.3, −0.09)	0.04	0.27
cg24998879	6			0.49 (0.02, 0.96)	0.04	0.28
cg11841722	5	TSS1500;5′UTR	*LIFR*	0.93 (0.04, 1.83)	0.04	0.28
cg04435320	11	Body	*TCP11L1*	0.58 (0.02, 1.13)	0.04	0.28
cg17160984	10			−1.67 (−3.28, −0.06)	0.04	0.28
cg11798873	6	Body	*PTPRK*	0.58 (0.01, 1.15)	0.05	0.32

CI, confidence interval; ^a^ gene region annotation of CpG probe position (Body (gene body), TSS200 (200 bp from transcription start site), TSS1500 (1500 bp from transcription start site), 5’UTR (5’ untranslated region), 1stExon (first exon)); ^b^ average change in CpG methylation under the variable of interest (β estimate) with a 95% confidence interval (CI); ^c^ Benjamani–Hochberg (BH) adjusted *p*-values; * *q* ≤ 0.05.

**Table 4:** Prenatal umbilical cord levels of metal mixtures and placental epigenetic gestational age acceleration (eGAA).

Metal Group	Population	Crude β (95% CI)	Adjusted β (95% CI)	Adjusted Positive Weights	Adjusted Negative Weights
All Metals	Full	−0.09 (−0.29, 0.11)	−0.02 (−0.23, 0.19)	Se: 0.27 Sr: 0.27 Sb: 0.20 Cd: 0.17 Ba: 0.06 Zn: 0.02 Hg: 0.01	Cu: −0.47 Mn: −0.30 Pb: −0.13 As: −0.10
All Metals	Female	−0.07 (−0.43, 0.29)	−0.07 (−0.45, 0.31)	Sr: 0.25 Cu: 0.20 As: 0.17 Hg: 0.17 Mn: 0.14 Cd: 0.05 Zn: 0.02	Se: −0.46 Ba: −0.26 Pb: −0.20 Sb: −0.08
All Metals	Male	−0.10 (−0.33, 0.13)	−0.03 (−0.28, 0.22)	Se: 0.33 Ba: 0.25 Pb: 0.21 Sb: 0.13 Zn: 0.08	Mn: −0.33 Cu: −0.32 Hg: −0.16 As: −0.13 Cd: −0.04 Sr: −0.02
Essential Metals	Full	−0.11 (−0.27, 0.05)	−0.09 (−0.26, 0.08)	Se: 0.73 Zn: 0.27	Cu: −0.58 Mn: −0.42
Essential Metals	Female	−0.05 (−0.30, 0.20)	−0.05 (−0.33, 0.23)	Cu: 0.67 Zn: 0.29 Mn: 0.04	Se: −1.00
Essential Metals	Male	−0.13 (−0.32, 0.06)	−0.11 (−0.34, 0.12)	Se: 0.92 Zn: 0.08	Cu: −0.50 Mn: −0.50
Non-Essential Metals	Full	−0.04 (−0.20, 0.13)	0.05 (−0.14, 0.24)	Sr: 0.54 Cd: 0.28 Sb: 0.18	Pb: −0.49 As: −0.33 Hg: −0.14 Ba: −0.04
Non-Essential Metals	Female	0 (−0.26, 0.26)	−0.04 (−0.34, 0.26)	Sr: 0.61 Hg: 0.20 As: 0.19	Ba: −0.56 Pb: −0.33 Sb: −0.09 Cd: −0.02
Non-Essential Metals	Male	−0.04 (−0.24, 0.16)	0.12 (−0.13, 0.37)	Ba: 0.46 Pb: 0.36 Sb: 0.16 Sr: 0.02	Hg: −0.69 As: −0.16 Cd: −0.16

CI, confidence interval.

## Data Availability

The data presented in this study are available on request from the corresponding author.

## Supplementary Materials

The following supporting information can be downloaded at https://www.mdpi.com/article/10.3390/cells14040306/s1: Table S1: Spearman’s rank correlation test coefficients between pairs of the 11 metals (antimony (Sb), arsenic (As), barium (Ba), cadmium (Cd), copper (Cu), lead (Pb), manganese (Mn), mercury (Hg), selenium (Se), strontium (Sr), zinc (Zn)) from umbilical cords examined in the Extremely Low for Gestational Age Newborn (ELGAN) study; Table S2: Distributions of umbilical cord metal concentrations in the Extremely Low for Gestational Age Newborn (ELGAN) study analytic sample (n = 265) by New England, North Carolina, and Midwest regions; Table S3: Associations between individual prenatal metal exposure and placental epigenetic gestational age acceleration (eGAA) stratified by New England, North Carolina, and Midwest regions in the Extremely Low for Gestational Age Newborn (ELGAN) study; Table S4: Prenatal umbilical cord levels of metal mixtures and placental epigenetic gestational age acceleration (eGAA) stratified by New England, North Carolina, and Midwest regions in the Extremely Low for Gestational Age Newborn (ELGAN) study; Figure S1: Study directed acyclic graph (DAG); Figure S2: Spearman correlation between chronological gestational age (GA) and epigenetic gestational age (eGA) estimated by the Robust Placental Clock (RPC) in the overall sample (n = 265), female infants (n = 120), and male infants (n = 145); Figure S3: Associations between Extremely Low for Gestational Age Newborn (ELGAN) study umbilical cord metal at a given quartile and placental epigenetic gestational age acceleration (eGAA).
