# Supplementary material for: Prenatal Exposure to Metals Is Associated with Placental Decelerated Epigenetic Gestational Age in a Sex-Dependent Manner in Infants Born Extremely Preterm
Source: Cells. 2025 Feb 18;14(4):306. doi: 10.3390/cells14040306 (PMC11854159; doi:10.3390/cells14040306)
Supplement: Supplementary file 1 [file cells-14-00306-s001.zip › supplement.pdf]

SUPPLEMENTAL TABLES & FIGURES:
"Prenatal Exposure to Metals Is Associated with Placental Decelerated Epigenetic Gestational Age in a Sex-Dependent Manner in Infants Born Extremely Preterm"

Table S1. Spearman's rank correlation test coefficients between pairs of the 11 metals (antimony (Sb), arsenic (As), barium (Ba), cadmium (Cd), copper (Cu), lead (Pb), manganese (Mn), mercury (Hg), selenium (Se), strontium (Sr), zinc (Zn)) from umbilical cord examined in the Extremely Low for Gestational Age Newborn (ELGAN) study.

Metal 1	Metal 2	Spearman Coefficient	*p*
Cu (μg/g)	Mn (μg/g)	0.134	0.029
Zn (μg/g)	Mn (μg/g)	0.190	0.002
As (ng/g)	Mn (μg/g)	0.124	0.044
Se (μg/g)	Mn (μg/g)	0.261	0.000
Sr (μg/g)	Mn (μg/g)	0.050	0.419
Cd (ng/g)	Mn (μg/g)	0.091	0.141
Sb (ng/g)	Mn (μg/g)	0.144	0.019
Ba (ng/g)	Mn (μg/g)	0.243	0.000
Hg (ng/g)	Mn (μg/g)	0.144	0.019
Pb (ng/g)	Mn (μg/g)	0.126	0.041
Zn (μg/g)	Cu (μg/g)	0.136	0.027
As (ng/g)	Cu (μg/g)	0.122	0.047
Se (μg/g)	Cu (μg/g)	0.223	0.000
Sr (μg/g)	Cu (μg/g)	0.100	0.106
Cd (ng/g)	Cu (μg/g)	0.133	0.031
Sb (ng/g)	Cu (μg/g)	0.121	0.050
Ba (ng/g)	Cu (μg/g)	0.133	0.030
Hg (ng/g)	Cu (μg/g)	0.107	0.083
Pb (ng/g)	Cu (μg/g)	0.270	0.000
As (ng/g)	Zn (μg/g)	0.364	0.000
Se (μg/g)	Zn (μg/g)	0.345	0.000
Sr (μg/g)	Zn (μg/g)	0.216	0.000
Cd (ng/g)	Zn (μg/g)	0.383	0.000
Sb (ng/g)	Zn (μg/g)	0.200	0.001
Ba (ng/g)	Zn (μg/g)	0.267	0.000
Hg (ng/g)	Zn (μg/g)	0.273	0.000
Pb (ng/g)	Zn (μg/g)	0.285	0.000
Se (μg/g)	As (ng/g)	0.108	0.079
Sr (μg/g)	As (ng/g)	0.229	0.000
Cd (ng/g)	As (ng/g)	0.300	0.000
Sb (ng/g)	As (ng/g)	0.262	0.000

SUPPLEMENTAL TABLES & FIGURES:
"Prenatal Exposure to Metals Is Associated with Placental Decelerated Epigenetic Gestational Age in a Sex-Dependent Manner in Infants Born Extremely Preterm"

Ba (ng/g)	As (ng/g)	0.172	0.005
Hg (ng/g)	As (ng/g)	0.498	0.000
Pb (ng/g)	As (ng/g)	0.313	0.000
Sr (μg/g)	Se (μg/g)	-0.018	0.770
Cd (ng/g)	Se (μg/g)	0.081	0.188
Sb (ng/g)	Se (μg/g)	-0.154	0.012
Ba (ng/g)	Se (μg/g)	-0.082	0.185
Hg (ng/g)	Se (μg/g)	0.099	0.106
Pb (ng/g)	Se (μg/g)	0.055	0.372
Cd (ng/g)	Sr (μg/g)	0.141	0.022
Sb (ng/g)	Sr (μg/g)	0.217	0.000
Ba (ng/g)	Sr (μg/g)	0.489	0.000
Hg (ng/g)	Sr (μg/g)	0.092	0.134
Pb (ng/g)	Sr (μg/g)	0.188	0.002
Sb (ng/g)	Cd (ng/g)	0.223	0.000
Ba (ng/g)	Cd (ng/g)	0.348	0.000
Hg (ng/g)	Cd (ng/g)	0.122	0.047
Pb (ng/g)	Cd (ng/g)	0.390	0.000
Ba (ng/g)	Sb (ng/g)	0.451	0.000
Hg (ng/g)	Sb (ng/g)	0.200	0.001
Pb (ng/g)	Sb (ng/g)	0.313	0.000
Hg (ng/g)	Ba (ng/g)	0.115	0.061
Pb (ng/g)	Ba (ng/g)	0.320	0.000
Pb (ng/g)	Hg (ng/g)	0.392	0.000

SUPPLEMENTAL TABLES & FIGURES:
"Prenatal Exposure to Metals Is Associated with Placental Decelerated Epigenetic Gestational Age in a Sex-Dependent Manner in Infants Born Extremely Preterm"

Table S2. Distributions of umbilical cord metals concentrations in the Extremely Low for Gestational Age Newborn (ELGAN) study analytic sample (*n*=265) by New England, North Carolina, and Midwest regions.

Metal	Unit	Median (IQR)			p^1
		New England (*n*=120)	North Carolina (*n*=81)	Midwest (*n*=64)	
Copper (Cu)	µg/g	3.59 (3.09, 4.51)	3.28 (2.88, 3.94)	3.59 (2.91, 4.27)	n.s.
Manganese (Mn)	µg/g	0.37 (0.31, 0.42)	0.32 (0.28, 0.43)	0.33 (0.29, 0.41)	n.s.
Selenium (Se)	µg/g	0.87 (0.80, 0.97)	0.78 (0.70, 0.85)	0.93 (0.86, 1.01)	**
Zinc (Zn)	µg/g	63.50 (56.49, 79.78)	51.00 (44.90, 58.09)	64.40 (55.93, 76.68)	**
Antimony (Sb)	ng/g	3.78 (2.32, 6.61)	2.71 (1.80, 5.58)	2.27 (1.29, 4.10)	**
Arsenic (As)	ng/g	5.85 (4.12, 9.79)	3.32 (2.32, 5.13)	4.65 (3.26, 6.22)	**
Barium (Ba)	ng/g	90.80 (65.37, 148.90)	71.40 (47.60, 101.99)	81.05 (52.88, 125.98)	*
Cadmium (Cd)	ng/g	1.55 (0.86, 3.51)	1.03 (0.69, 1.93)	1.36 (0.65, 3.70)	*
Lead (Pb)	ng/g	23.10 (16.55, 42.95)	11.20 (7.20, 19.20)	10.05 (6.03, 15.90)	**
Mercury (Hg)	ng/g	14.93 (8.42, 29.71)	6.19 (3.29, 8.92)	4.11 (2.23, 9.06)	**
Strontium (Sr)	ng/g	0.57 (0.40, 0.90)	0.47 (0.35, 0.64)	0.64 (0.46, 0.93)	**

IQR, interquartile range from the 25th percentile to 75th percentile
[1]Kruskal-wallis rank sum test comparing median metal concentrations among regions
*$p<0.05$
**$p<0.001$

SUPPLEMENTAL TABLES & FIGURES:
"Prenatal Exposure to Metals Is Associated with Placental Decelerated Epigenetic Gestational Age in a Sex-Dependent Manner in Infants Born Extremely Preterm"

Table S3. Associations between individual prenatal metal exposure and placental epigenetic gestational age acceleration (eGAA) stratified by New England, North Carolina, and Midwest regions in the Extremely Low for Gestational Age Newborn (ELGAN) study.

Metal	Unit	New England (*n*=120)		North Carolina (*n*=81)		Midwest (*n*=64)	
		Estimate (95% CI)	*p*	Estimate (95% CI)	*p*	Estimate (95% CI)	*p*
Copper (Cu)	µg/g	-0.85 (-2.04, 0.33)	0.16	0.59 (-0.94, 2.13)	0.44	-0.20 (-1.63, 1.22)	0.77
Manganese (Mn)	µg/g	-0.94 (-2.04, 0.15)	0.09	0.58 (-0.49, 1.65)	0.28	-0.06 (-1.21, 1.08)	0.91
Selenium (Se)	µg/g	-0.42 (-2.34, 1.50)	0.66	1.16 (-1.23, 3.54)	0.34	1.19 (-1.52, 3.91)	0.38
Zinc (Zn)	µg/g	0.33 (-0.60, 1.26)	0.49	-0.14 (-2.18, 1.89)	0.89	0.21 (-0.94, 1.37)	0.71
Antimony (Sb)	ng/g	0.22 (-0.12, 0.56)	0.19	0.01 (-0.47, 0.49)	0.96	-0.06 (-0.60, 0.48)	0.82
Arsenic (As)	ng/g	-0.27 (-0.86, 0.32)	0.37	0.18 (-0.57, 0.93)	0.63	0.11 (-0.47, 0.69)	0.70
Barium (Ba)	ng/g	-0.10 (-0.63, 0.44)	0.72	0.46 (-0.26, 1.17)	0.20	-0.12 (-0.68, 0.45)	0.68
Cadmium (Cd)	ng/g	-0.04 (-0.28, 0.20)	0.76	0.51 (-0.01, 1.04)	0.06	-0.21 (-0.50, 0.08)	0.15
Lead (Pb)	ng/g	-0.36 (-0.77, 0.05)	0.09	0.05 (-0.45, 0.56)	0.84	-0.31 (-0.97, 0.35)	0.34
Mercury (Hg)	ng/g	-0.05 (-0.44, 0.33)	0.78	-0.15 (-0.64, 0.34)	0.54	0.04 (-0.35, 0.44)	0.82
Strontium (Sr)	ng/g	-0.12 (-0.74, 0.51)	0.71	-0.12 (-0.99, 0.76)	0.79	0.65 (-0.06, 1.36)	0.07

CI, confidence interval

SUPPLEMENTAL TABLES & FIGURES:
"Prenatal Exposure to Metals Is Associated with Placental Decelerated Epigenetic Gestational Age in a Sex-Dependent Manner in Infants Born Extremely Preterm"

Table S4. Prenatal umbilical cord levels metal mixtures and placental epigenetic gestational age acceleration (eGAA) stratified by New England, North Carolina, and Midwest regions in the Extremely Low for Gestational Age Newborn (ELGAN) study.

Metal Group	Region	Crude β (95% CI)	Adjusted β (95% CI)	Adjusted Positive Weights	Adjusted Negative Weights
All Metals	New England	-0.44 (-0.75, -0.13)*	-0.30 (-0.59, -0.01)*	Sb: 0.48<br>Se: 0.28<br>Hg: 0.12<br>Sr: 0.08<br>Zn: 0.06	Mn: -0.26<br>Cu: -0.25<br>As: -0.22<br>Pb: -0.17<br>Cd: -0.08<br>Ba: -0.02
All Metals	North Carolina	0.19 (-0.18, 0.56)	0.10 (-0.29, 0.48)	Cd: 0.47<br>Ba: 0.41<br>Se: 0.08<br>Sr: 0.03<br>As: 0.01	Pb: -0.33<br>Mn: -0.26<br>Zn: -0.19<br>Hg: -0.10<br>Sb: -0.07<br>Cu: -0.05
All Metals	Midwest	0.35 (-0.05, 0.74)	0.17 (-0.22, 0.56)	Sr: 0.50<br>Se: 0.31<br>Zn: 0.09<br>As: 0.07<br>Sb: 0.03	Ba: -0.38<br>Cd: -0.32<br>Mn: -0.21<br>Pb: -0.07<br>Hg: -0.02<br>Cu: -0.00
Essential Metals	New England	-0.29 (-0.53, -0.06)*	-0.22 (-0.45, 0.02)	Se: 0.61	Mn: -0.54

SUPPLEMENTAL TABLES & FIGURES:
"Prenatal Exposure to Metals Is Associated with Placental Decelerated Epigenetic Gestational Age in a Sex-Dependent Manner in Infants Born Extremely Preterm"

				Zn: 0.39	Cu: -0.46
Essential Metals	North Carolina	0.08 (-0.18, 0.34)	-0.21 (-0.51, 0.09)	Se: 1.00	Mn: -0.54<br>Zn: -0.38<br>Cu: -0.08
Essential Metals	Midwest	0.16 (-0.14, 0.45)	0.23 (-0.09, 0.54)	Se: 0.72<br>Zn: 0.14<br>Cu: 0.13	Mn: -1.00
Non-Essential Metals	New England	-0.27 (-0.53, 0.00)*	-0.12 (-0.41, 0.17)	Sb: 0.75<br>Sr: 0.16<br>Hg: 0.09	As: -0.43<br>Pb: -0.29<br>Cd: -0.17<br>Ba: -0.10
Non-Essential Metals	North Carolina	0.12 (-0.18, 0.42)	0.34 (-0.05, 0.74)	Cd: 0.48<br>Ba: 0.37<br>Sr: 0.12<br>As: 0.04	Pb: -0.82<br>Hg: -0.13<br>Sb: -0.06
Non-Essential Metals	Midwest	0.14 (-0.16, 0.44)	0.07 (-0.31, 0.44)	Sr: 0.78<br>As: 0.13<br>Sb: 0.10	Ba: -0.42<br>Cd: -0.39<br>Pb: -0.14<br>Hg: -0.05

CI, confidence interval
*$p<0.05$

SUPPLEMENTAL TABLES & FIGURES:
"Prenatal Exposure to Metals Is Associated with Placental Decelerated Epigenetic Gestational Age in a Sex-Dependent Manner in Infants Born Extremely Preterm"

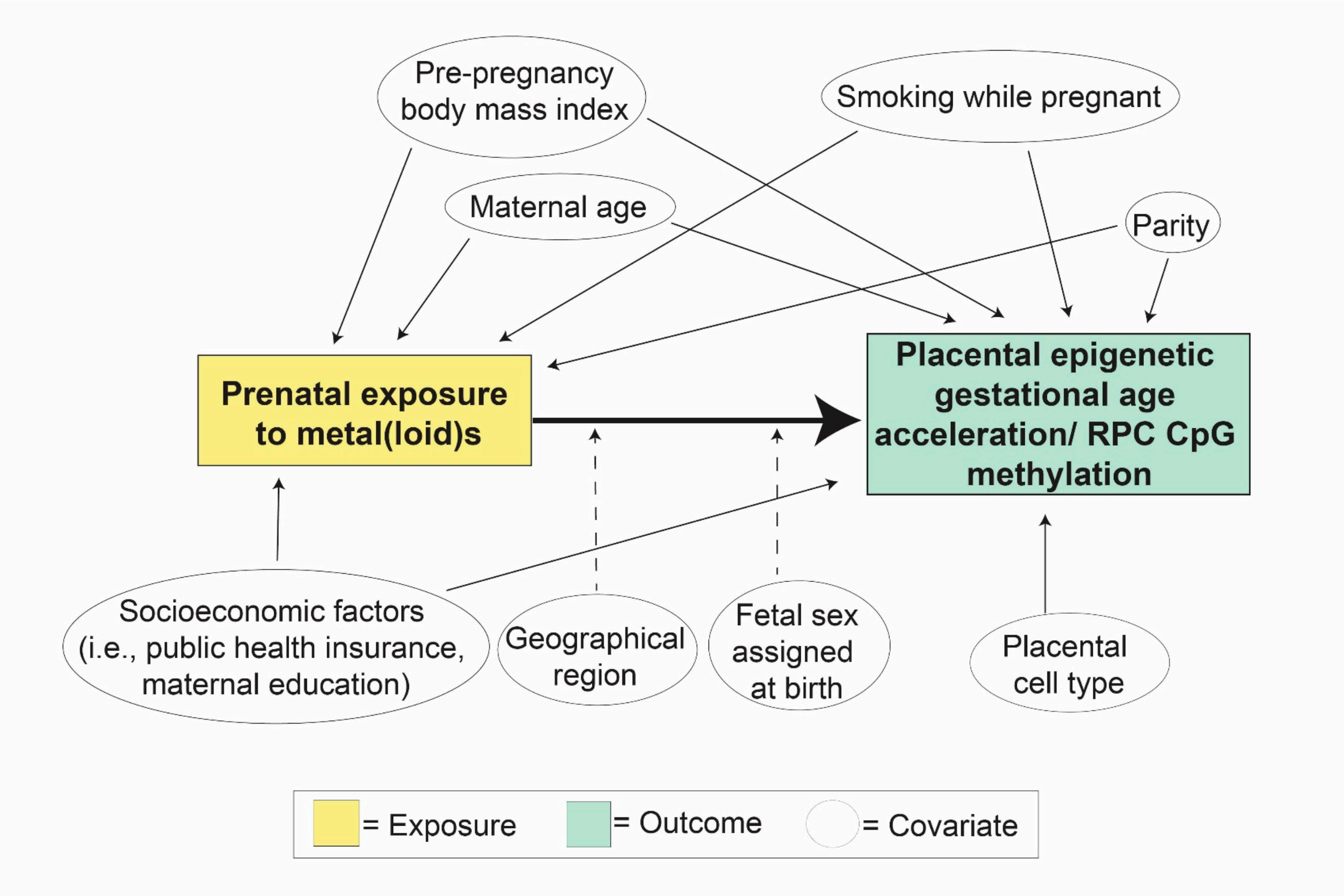

Figure S1. Study directed acyclic graph (DAG). This DAG demonstrates the minimally sufficient adjustment set of covariates used in this study's models.

SUPPLEMENTAL TABLES & FIGURES:
"Prenatal Exposure to Metals Is Associated with Placental Decelerated Epigenetic Gestational Age in a Sex-Dependent Manner in Infants Born Extremely Preterm"

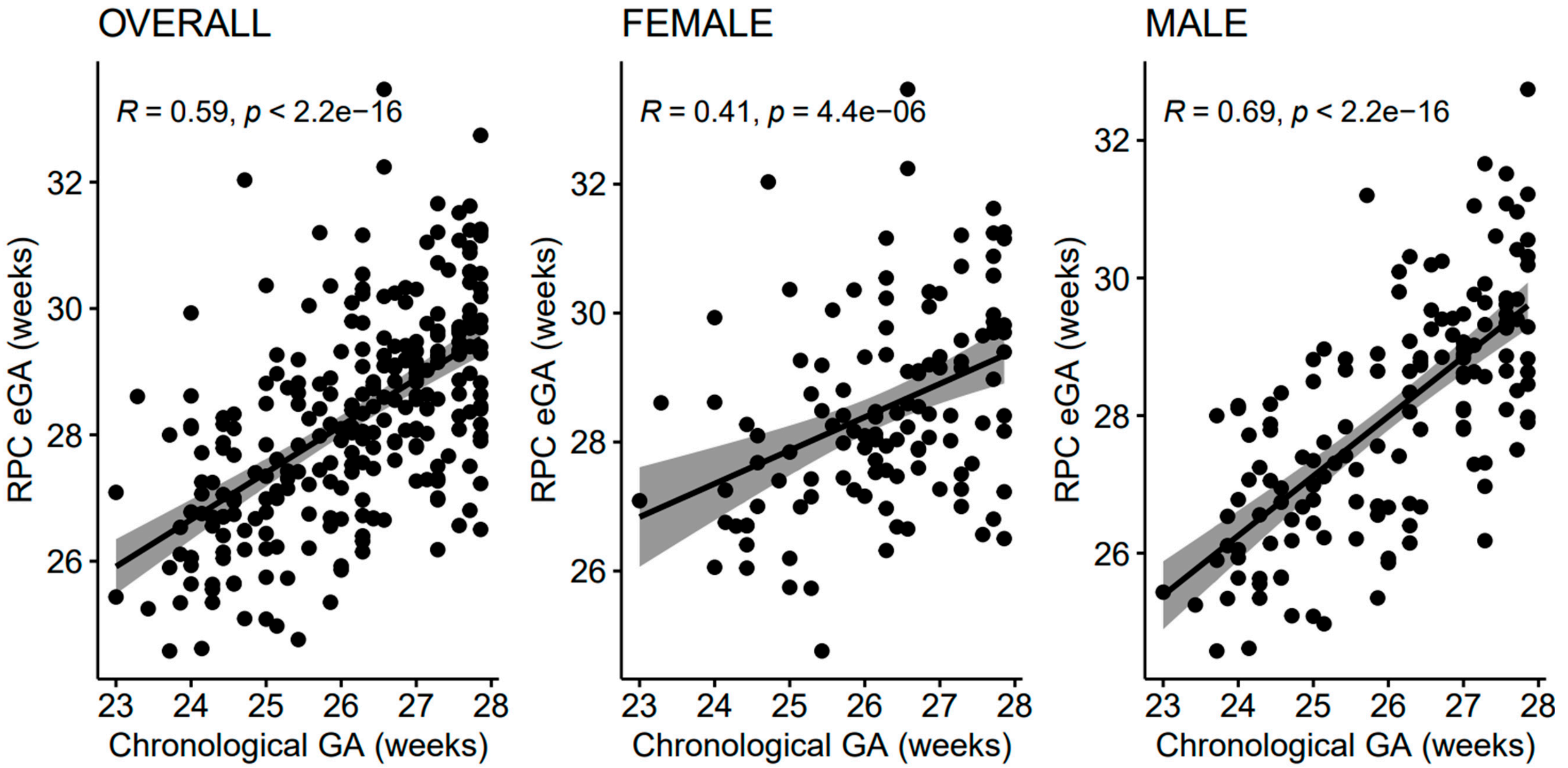

Figure S2. Spearman correlation between chronological gestational age (GA) and epigenetic gestational age (eGA) estimated by the Robust Placental Clock (RPC) in the overall sample (*n*=265), female infants (*n*=120), and male infants (*n*=145). The RPC accurately estimated GA in all groups with the strongest correlation in males (r=0.69, p<0.01, median absolute error (MAE)=1.99). Chronological GA moderately correlated with eGA in females (r=0.41, p<0.01, MAE=2.12) and the overall sample (r=0.59, p<0.01, MAE=2.04).

SUPPLEMENTAL TABLES & FIGURES:
"Prenatal Exposure to Metals Is Associated with Placental Decelerated Epigenetic Gestational Age in a Sex-Dependent Manner in Infants Born Extremely Preterm"

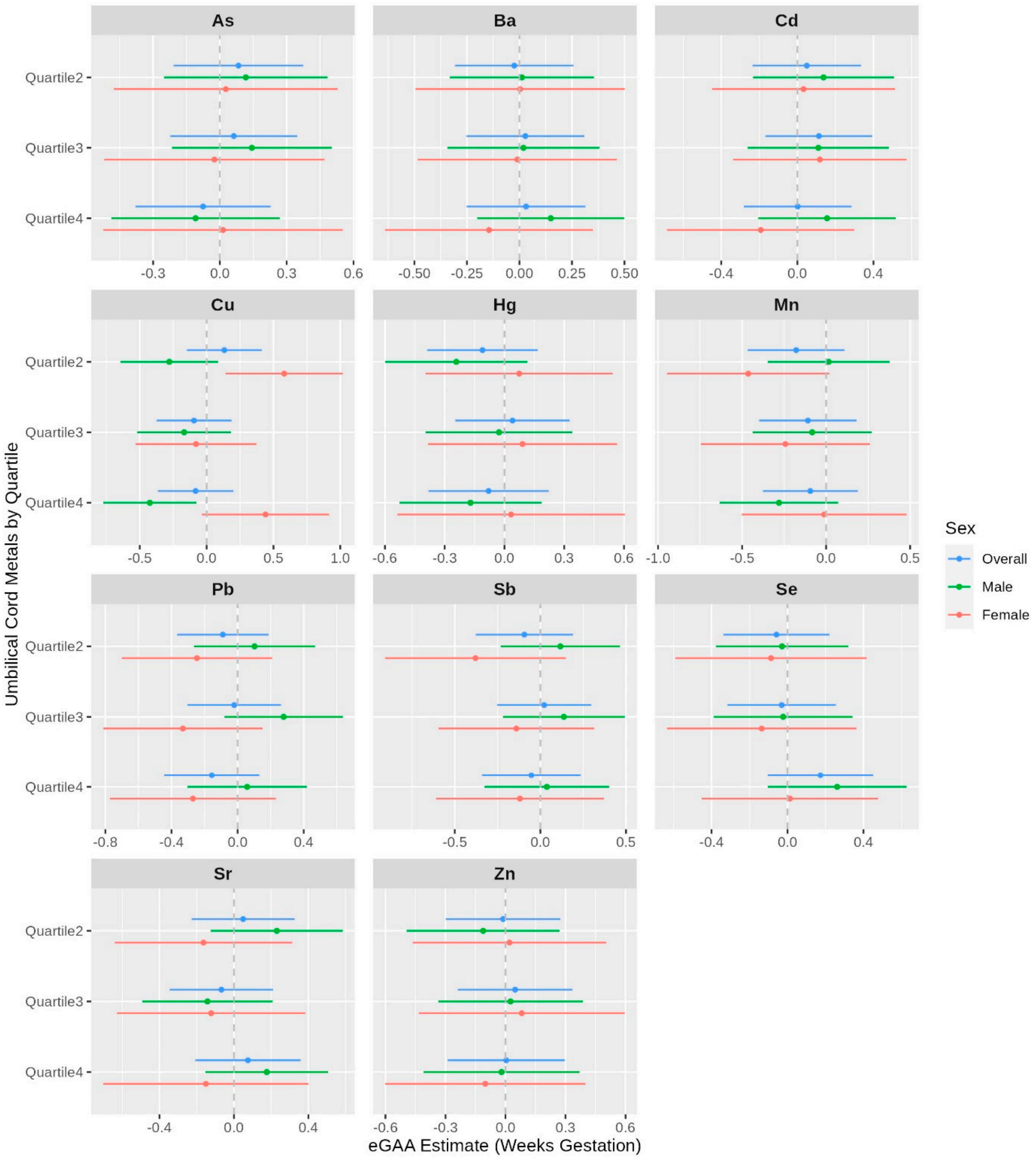

Figure S3. Associations between Extremely Low for Gestational Age Newborn (ELGAN) study umbilical cord metal at a given quartile and placental epigenetic gestational age acceleration (eGAA). Metal distributions were split into equal quartiles, with the first quartile designated as the reference value for each respective analysis, and

analyses were stratified by fetal sex. Blue denotes the full sample; green denotes only males; and red denotes only females.
